# Efficient Energy Supply Using Mobile Charger for Solar-Powered Wireless Sensor Networks

**DOI:** 10.3390/s19122679

**Published:** 2019-06-13

**Authors:** Jun Min Yi, Ikjune Yoon

**Affiliations:** Department of Smart Systems Software, Soongsil University, Seoul 06978, Korea; jmyi@ssu.ac.kr

**Keywords:** wireless sensor networks, rechargeable, energy-harvesting, mobile sink, drone

## Abstract

An energy-harvesting wireless sensor network mitigates the energy shortage problems of existing battery-based wireless sensors; however, its hotspot area sensor nodes still experience 3 blackouts, thereby reducing network connectivity. Techniques that transfer energy directly to sensor nodes using wireless power transfer (WPT) have been studied in recent years to address this issue. In this paper, we propose a technique that uses a drone (quadcopter), which is a type of unmanned aerial vehicle (UAV), as a mobile sink. The drone selects and manages anchor nodes that aggregate data temporarily, collects data by visiting the anchor nodes to mitigate the hotspot issue, and then prevents blackouts by supplying energy to low-energy nodes, thereby improving network connectivity. The anchor nodes are carefully selected after considering the energy capacity of the drone, the size of the network, the amount of collected data, and the energy consumed by the nodes to increase the network’s energy efficiency. Furthermore, energy is transferred from the drone to the anchor nodes to support their energy consumption. In our study, this method reduced the blackouts of sensor nodes, including anchor nodes, in hotspot regions, and increased network connectivity, thereby improving the amount of data gathered by the mobile sink.

## 1. Introduction

Recently, the further development of communication technology, including big data and the Internet of Things, has enabled a hyper-connected society. Data collection is a very important factor in such a society, and accordingly, the development of wireless sensor networks (WSNs) is becoming crucial. WSNs can be used to collect data in hazardous areas, such as volcanoes, during natural or man-made disasters, in farmlands, forests, and underwater, where wide areas must be monitored, in various industries to monitor structures including buildings and bridges for example, or in health and medical monitoring related to human health [1,2,3,4]. As the demand for WSNs grows, corresponding technologies are also evolving. However, problems regarding (i) the limited network lifetime of battery-operated sensor nodes and (ii) hotspots formed when a large volume of packets are sent from the sensor nodes of a small area, causing an obvious inconsistency in the network traffic [5], have not been solved.

Developing the methods to address these issues is becoming a major research area. WSNs consist of small devices that are low-cost and battery-based, and large numbers of these are installed in large areas to collect data. In traditional WSNs, to maintain network connectivity, either a sensor node’s battery or the node itself must be replaced frequently. However, there are many difficulties and limitations when a WSN relies on manual maintenance of multiple sensor nodes, and it is also costly to do so. To overcome these problems, techniques to minimize energy consumption have been actively studied [6,7,8].

Using energy-harvesting sensor nodes is one method to solve the limited energy problem of battery-powered sensor nodes. Energy-harvesting sensor nodes collect energy from available sources, such as the sun or wind [9,10], and theoretically, permanent operation is possible unless a hardware failure occurs. In particular, solar energy is preferred because of its high energy density [11]. However, this type of energy is limited by the amount and timing of the energy that can be collected. Therefore, it is difficult to continuously supply the required energy needed to operate the sensor nodes.

In recent years, wireless power transfer (WPT) technology, which transmits energy across various distances, has been introduced and used in various fields [12]. There are three main methods of implementing WPT: inductive coupling, magnetic resonance coupling, and radio frequency (RF). Each method has different characteristics, such as transmission distance, charging efficiency, and the effects of obstacles [13,14].

In WSNs as well as other fields, WPT has been extensively studied to solve the limited energy problem. Researchers have used fixed antennas to supply energy wirelessly to sensor nodes, or have installed charging equipment in automobiles and unmanned aerial vehicles to generate energy while the vehicles are moving [12,15], and other research has been actively conducted to increase the efficiency of the energy supply [14,16,17]. Compared with an automobile, where it is possible to supply sufficient energy without major restrictions on the weight or size of the charging devices, there are many restrictions affecting the movement of a drone, thus limiting its operating environment. Meanwhile, unlike automobiles, a UAV can move without any restrictions based on the type of terrain, but its weight-carrying capacity, battery size, and range of transmission are limited, thereby limiting its flight time, and hence requiring efficient routing and battery management schemes [15,18].

The second problem with WSNs is the frequent blackout of sensor nodes in areas where a large amount of data is transferred in a short time, which is a characteristic of multi-hop transmissions. Theoretically, a method using a mobile sink can solve the problem. Generally, in WSNs, data is collected using a sink node of a fixed facility such as a base station, but the mobile sink moves to sensor nodes and collects data of the corresponding nodes. In reality, however, it is not possible for a sink to visit hundreds or even thousands of sensor nodes to collect data, and even if it were feasible, it would be impractical and very inefficient. Therefore, only a few nodes are approached to collect accumulated data. Typically, the nodes closer to a data-aggregating node consume more energy than other nodes; consequently, blackouts can easily occur, resulting in large data losses. Additionally, when some nodes black out, their loads are transferred to their neighboring nodes, causing blackouts in those nodes as well, thereby making the network inoperable. To solve this problem, techniques such as hierarchical topology, clustering, data compression, and energy-aware routing have been studied [19,20,21].

Despite these studies, because of the differences between the energy used by battery-based and energy-harvesting WSNs, the traditional techniques are not suitable for energy-harvesting WSNs. To increase network lifetime and performance in energy-harvesting WSNs, suitable strategies for efficient energy collection, consumption, and supply need to be developed.

In this paper, we propose a scheme where a drone with WPT capability is used as a mobile sink to select and manage anchor nodes that aggregate data temporarily to mitigate hotspot problems and improve network connectivity in an energy-harvesting WSN. Anchor nodes are selected and managed by considering the drone’s energy capacity, data collection capacity, and energy consumption to improve the network connectivity and the amount of data collected by efficiently using the limited energy of the drone. The mobile sink (the drone) visits the anchor nodes and collects the aggregated data to prevent heavy loads in hotspot areas, and simultaneously distributes usable energy to each anchor node, which consumes more energy than other nodes. This prevents node blackouts, thereby improving the connectivity of the network.

This paper is organized as follows. In Section 2, the schemes related to energy harvesting, WPT, and mobile sinks are introduced. In Section 3, we describe in detail how to operate the mobile sink and anchor nodes. In Section 4, the performance evaluation of the proposed scheme is described, and finally, a conclusion is presented in Section 5.

## 2. Related Work

In this section, we review schemes for wireless power transfer and mobile sink use for WSNs.

### 2.1. Wireless Power Transfer for WSN

WPT is a technique that transmits energy to a device wirelessly; it has been used recently in environment where it is difficult to connect devices by using wires. There are three WPT-based wireless charging methods, as discussed earlier: inductive coupling, magnetic resonance coupling, and RF. The advantage of inductive coupling is in enabling high power transmission through electric induction between the primary and secondary coils of a transformer; its transmission distance is less than a few mm and its efficiency is more than 90%. In magnetic resonance coupling, the resonance between transmitting and receiving antennas is used, which requires large antennas, and high power transmission is difficult. The efficiency is 90% at a distance of 1 m and declines to 40% at a distance of 2 m. In the RF methods, electromagnetic waves are directly transmitted and received through an antenna, which can be affected by obstacles, but it is possible to transmit up to tens of km with an efficiency ranging from 10% to 50%.

Lu et al. [13] proposed a circuit design method and a communication protocol for WPT. They proposed design considerations based on the type of network. In addition, they discussed the problems that arise when integrating wireless charging with a wireless communication system, the development of wireless charging technology standards, and their applications. They also discussed charger scheduling, dispatch, and deployment strategies for network applications [22]. Kurs et al. [23] proposed a stable energy transfer technology in which self-resonant coils were manufactured to transmit 60 watts at 40% energy efficiency over a 2 m distance, and they proposed a method to transmit energy simultaneously to several receivers by magnetic resonance coupling [24].

Several studies applying WPT to WSNs have overcome the limited energy of sensor nodes [12]. Sangare et al. [17] developed a prototype using off-the-shelf RF energy transmission equipment to verify the actual performance of RF energy delivery in WSNs. They further proposed a heuristic algorithm to supply energy efficiently by adjusting three parameters: the number of nodes, the distance between nodes, and the distance between a node and a charger. Li et al. [25] proposed an energy-harvesting Markov decision process (EHMDP) technique that charges a sensor node from the base station through WPT and minimizes the data packet loss of the sensor nodes by considering energy consumption and data queue status. Fang et al. [26] proposed a technique to control the amount of data transmission depending on the energy state during WPT.

WPT becomes less efficient as the transmission distance increases and it becomes more difficult to implement as the network size increases. As a method to solve this problem, studies that evaluate installing chargers in vehicles have been published. Guo et al. [27] proposed a WPT through use of a multi-functional mobile collector, namely SenCar and an anchor point-based mobile data collection framework. In this technique, the anchor point selection strategy and visiting order are determined by considering the battery capacity and energy balance of the network’s sensor nodes. Khelladi [28] proposed a minimum-stop recharging technique, which is a heuristic algorithm that optimizes the charging time of nodes by maximizing the number of nodes being charged simultaneously at the stop position of the mobile charger while minimizing the number of stop points. Fu et al. [14] proposed energy synchronized mobile charging (ESync), which is an energy synchronization technique that reduces the travel distance of a mobile charger. It reduced the travel distance by 30–40% from Nearest-Job-Next [29]. Liu et al. [30] proposed an optimal buffer-battery adaptive scheduling technique using the Lyapunov drift theory in a mobile sink-based WSN. Tu et al. [15] proposed an energy supply scheme using a car traversing a predefined path.

In large-scale (or difficult-to-access) WSNs, mobile chargers cannot transmit energy to all nodes by visiting them individually, so schemes using a combination of energy-harvesting and WPT are also being actively studied to compensate for this problem. Shruti et al. [31] proposed a device design using both solar cells and WPT. Har [32] proposed a method to supply energy from a RF mobile charger to energy-harvesting nodes through energy trading between nodes. This scheme uses a two-step RF charging method that uses a directional antenna to charge the cluster heads, and they in turn charge other sensor nodes. Wang et al. [33] proposed a three-tier topology management scheme that uses both wireless charging and energy-harvesting methods in a network comprised of energy-harvesting and battery-powered nodes. In this scheme, both solar energy and WPT are applied to meet the high energy requirements of nodes in the cluster heads.

The use of most of these techniques is difficult in terrain where fixed antennas cannot be used or where large networks cannot be accessed using automobiles. Therefore, a technique to transfer energy using a UAV, such as a drone, is necessary to increase the lifetime and efficiency of a network, especially in areas that cannot be accessed easily.

### 2.2. WSN Using Mobile Sink

To solve the hotspot problem, methods of data collection by visiting sensor nodes using a mobile sink have been studied [34,35,36,37,38]. Yun and Xia [34] proposed a scheme in which a mobile sink collects temporarily stored data from sensor nodes in the most advantageous positions so that the lifetime of the WSN can be extended. Their scheme can increase the network lifetime more effectively than the conventional mobile sink operating method or the fixed sink model. Ren et al. [35] designed a technique in which a mobile sink collects data while traversing a predefined path in an energy-harvesting WSN. Shin et al. [36] proposed a railroad architecture that makes all nodes easily accessible by setting up a rail in the central region of the network field. When a node senses an event, it sends data along the rail, reducing energy consumption in a hotspot area. Berrahal et al. [37] proposed a border monitoring solution using a drone. By using the drone as a mobile sink, it can detect coverage holes, identify and investigate faults, deliver urgent data, and capture real-time video in the field. However, improvements are needed because of the limited energy of the mobile sink. Tazibt et al. [20] proposed a scheme in which cluster heads aggregate data and a UAV visits them to collect the data. The cluster heads are determined by considering the maximum number of hops from the sensor nodes to the cluster heads. This results in reduced energy consumption of the sensor nodes and extends the network’s lifetime.

The studies mentioned above do not consider the case where wireless charging needs to be performed alongside data collection, because these studies have focused only on data collection. Therefore, research to increase the lifetime of the network and increase data collection by using a mobile sink that performs wireless charging and data collection simultaneously is of interest.

## 3. Wireless Power Transfer and Data Collection Using Mobile Sink

**Figure 1:**
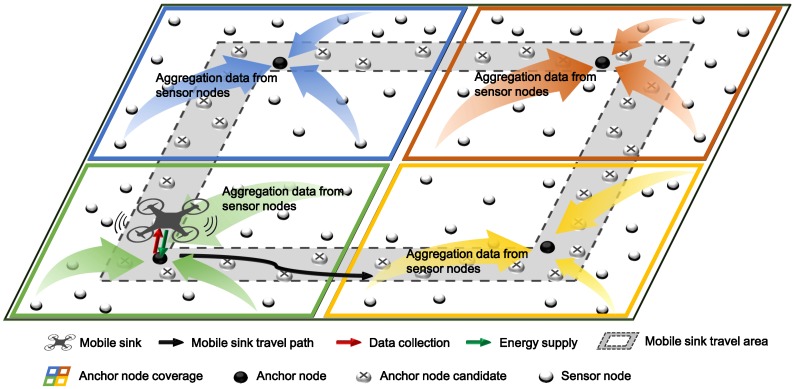
Overview of the proposed scheme.

In this paper, we propose a scheme to mitigate energy shortages in hotspots and collect data efficiently by selection and management of anchor nodes in energy-harvesting WSNs using drones as mobile sinks and energy chargers simultaneously. The drone that has limited energy cannot visit all sensor nodes, anchor nodes temporarily collect data from other sensor nodes, and the mobile sink can collect data of the entire network by only visiting the anchor nodes. In this scheme, the anchor nodes are selected dynamically at runtime by the mobile sink at the beginning of a round, the sensor nodes transmit sensed data to anchor nodes periodically, and mobile sinks travel through predefined paths to receive the aggregated data from anchor nodes. Consequently, the anchor nodes consume more energy than the other sensor nodes, but the mobile sink supplies the energy needed so that anchor nodes do not suffer outages. The numbers and locations of the anchor nodes are dynamically determined by calculating the energy of the mobile sink and the node consumption before a mobile sink traverses the network to collect data, and the next anchor node is selected from the next anchor candidate area while the sink is traveling. This scheme is designed for a delay tolerant network in which transmission delay is not important, such as environmental monitoring, and data can be efficiently collected in subject areas that are inaccessible to automobiles or people, such as in volcanic areas, deep underwater, and isolated ecosystems. Figure 1 shows an overview of the proposed scheme.

### 3.1. Determination of a Mobile Sink’s Route

**Figure 2:**
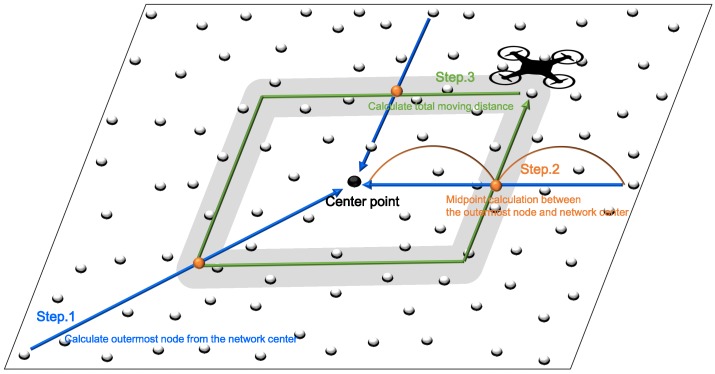
Selection of the traversal path of a mobile sink.

In WSNs using UAVs, it is difficult to collect data by visiting all nodes because the traveling distance of the UAV is limited by its battery life, especially when there are numerous sensor nodes. Therefore, many WSN applications restrict the paths mobile sinks traverse. In this study, a mobile sink collects data by visiting anchor nodes situated only along a predefined path. The path used in this technique is the same as the *railroad* method proposed by Shin et al. [36]. *Railroad* is a data dissemination architecture for large-scale WSN. *Railroad* system proactively exploits a virtual infrastructure called *rail*, which is an area where all the metadata of event data are stored, and the rail acts as a rendezvous area of several data. We choose the middle point between the outermost node of the network field and the center point of the field as the travel path of the drone similar to the *rail* to ensure that all nodes can be accessed equally and where the number of hops between the mobile sink and sensor nodes is minimized as shown in Figure 2. The width of the path is set to be within the transmission range of the drone so that when the drone moves to the middle of this path, all nodes on this path can communicate with the drone directly. The drone moves clockwise or counterclockwise along this path. When it arrives at the anchor node or the anchor candidate area while moving, it collects data form the anchor node or executes the anchor selection process for the anchor candidate area. The anchor selection process is described in Section 3.3. In this process, other sensor nodes do not need to know their location except for the drone. The sensor nodes determine their role by communicating with the drone and transmit data forward the drone because the drone determines their path in consideration of the size of the network and Since the drone determines its own path considering the size of the network, and selects anchor nodes by traversing the path, other sensor nodes can determine their routes by communicating with the anchors and transmit the data along the routes. In this case, the drones can use the methods proposed by Oliva et al. [39] and Ji et al. [40] to obtain more precise location of the nodes to which the drone should transmit energy. Alternatively, the location and routing of nodes can be performed simultaneously using the scheme proposed by Oliva et al. [41].

However, no matter how large the network size, the path length of the drone cannot be longer than the maximum flight distance of the drone owing to the limited endurance of the drone. If the path of the mobile sink set as mentioned above is longer than the maximum flight distance, the mobile sink should change it to a short enough distance to travel. At this time, the mobile sink’s travel path must be set closer to the network center, allowing the mobile sink to successfully traverse the network. In this case, since the number of data transmission hops of the sensor nodes may be longer, the overall performance of the network can decrease. Therefore, we should reduce the number of hops using multiple drones or a method such as dividing the network into small areas when the network is large. In addition, when selecting the drone’s travel path and anchors in the above method, the number of nodes transmitting data to the anchors can be varied according to the location of anchors. For instance, if the shape of network field is a rectangle, the anchors at the corner of the rectangle can receive more data than other anchors. Conversely, if the network field is circular, all anchors receive the similar amount of data. We have determined the travel path of the drone in the similar method as the railroad scheme to support diverse networks fairly. Figure 2 shows how to determine the path for a mobile sink.

### 3.2. Energy Models

#### 3.2.1. Energy Model of a Sensor Node

To select the anchor node and determine the energy needed to maintain its charge, the energy state of the sensor node should be known. We calculate the amount of energy consumed and harvested for one round, in which a mobile sink traverses around the entire network or the sector of the network it has been assigned to. The sensor node consumes energy through data sensing and transmission for one round, and collects environmental energy to charge its battery. The remaining energy in the node ${\hat{e_{}}}_{\mathrm{remain}}$ in the next round can be calculated as follows:

(1) $${\hat{e_{}}}_{\mathrm{remain}}={e_{}}_{\mathrm{remain}}-{e_{}}_{c}+{e_{}}_{h},$$

where ${e_{}}_{\mathrm{remain}}$, ${e_{}}_{c}$, and ${e_{}}_{h}$ are the remaining energy, consumed energy, and collected energy, respectively. ${e_{}}_{\mathrm{remain}}$ can be measured in the sensor node, eh can be predicted through the weather-conditioned moving average (WCMA) proposed by Piorno et al. [42], the exponentially weighted moving average (EWMA) proposed by Cox [43], or the Pro-Energy proposed by Cammarano et al. [44].

${e_{}}_{c}$ can be estimated from ${e_{}}_{\mathrm{Tx}}$, which is the energy consumed when transmitting radio waves, ${e_{}}_{\mathrm{Rx}}$ which is the energy consumed when receiving data, and from ${e_{}}_{\mathrm{sys}}$, which is the energy consumed during standby and idle states. Therefore, ${e_{}}_{c}$ can be calculated as follows:

(2) $${e_{}}_{c}={e_{}}_{\mathrm{Tx}}+{e_{}}_{\mathrm{Rx}}+{e_{}}_{\mathrm{sys}}.$$

In this equation, ${e_{}}_{\mathrm{Rx}}$ and ${e_{}}_{\mathrm{sys}}$ can be known according to the configuration of the nodes because ${e_{}}_{\mathrm{Rx}}$ and ${e_{}}_{\mathrm{sys}}$ depend on the time node’s transceiver is turned on and the node’s operating time, respectively. ${e_{}}_{\mathrm{Tx}}$ can vary depending on the amount of data transmitted and the transmission distance. Based on the energy consumption model of Melodia et al. [45], this can be estimated as follows:

(3) $${e_{}}_{\mathrm{Tx}}=s\beta {r_{}}^{\alpha },$$

where *s* is the number of bytes of data to be transmitted, the constant β is the energy consumed by the transmission per byte according to the distance ($J/\mathrm{bytes}/m^{\mathrm{ff}}$), $r_{}$ is the transmission distance in meters, and α is the path loss exponent (2≤α≤5). By substituting Equations (2) and (3) into Equation (1), the estimated remaining energy in the next round, ${\hat{e_{}}}_{\mathrm{remain}}$ can be calculated as follows:

(4) $${\hat{e_{}}}_{\mathrm{remain}}={e_{}}_{\mathrm{remain}}-\left(s\beta {r_{}}^{\alpha }+{e_{}}_{\mathrm{Rx}}+{e_{}}_{\mathrm{sys}}\right)+{e_{}}_{h}.$$

**Figure 3:**
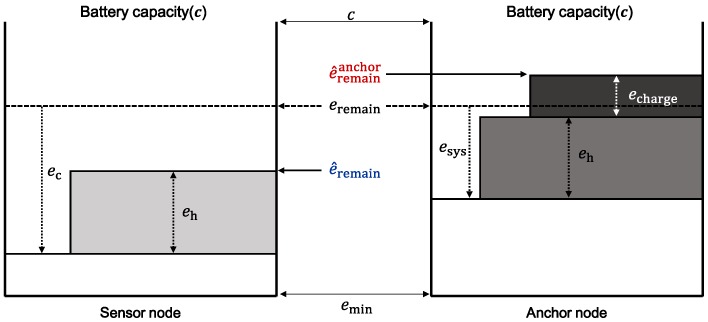
Energy model of a sensor node.

Figure 3 shows the simplified energy model of a sensor node.

#### 3.2.2. Energy Model of an Anchor Node

An anchor node aggregates data from sensor nodes and delivers aggregated data to the mobile sink when it arrives. Therefore, it transmits more data than other nodes and thus consumes more transmission energy ${e_{}}_{\mathrm{Tx}}$. However, the anchor node consumes less energy than other nodes adjacent to it until the mobile sink visits because it transmits data only if a sink arrives; otherwise, it only receives data. Therefore, the estimated remaining energy ${\hat{e_{}}}_{\mathrm{remain}}^{\mathrm{anchor}}$, excluding the data transfer part in Equation (4), of the anchor node in the next round, can be expressed as follows:

(5) $${\hat{e_{}}}_{\mathrm{remain}}^{\mathrm{anchor}}={e_{}}_{\mathrm{remain}}-{e_{}}_{\mathrm{Rx}}-{e_{}}_{\mathrm{sys}}+{e_{}}_{h}.$$

Therefore, if ${\hat{e_{}}}_{\mathrm{remain}}^{\mathrm{anchor}}$ is greater than the minimum energy required to operate the node, ${e_{}}_{\min}$, the anchor node can survive until the next round. In other words, if

(6) $${\hat{e_{}}}_{\mathrm{remain}}^{\mathrm{anchor}}\geq {e_{}}_{\min}$$

is satisfied, then the anchor can aggregate data and survive until the sink node arrives in the next turn. Conversely, the amount of harvested energy can be so high that it could exceed the capacity *c* of the battery in the node, and thus overcharge the battery. Therefore, the following condition:

(7) $${\hat{e_{}}}_{\mathrm{remain}}^{\mathrm{anchor}}\leq c_{}$$

should be satisfied so that no energy is wasted by overcharging the anchor node.

In this scheme, nodes whose energy is neither insufficient nor excessive are selected as anchor nodes by considering Equations (6) and (7). The detailed anchor selection process is described in Section 3.4.

#### 3.2.3. Energy Model of a Mobile Sink Node

The total energy of the mobile sink can be divided into the energy consumed by its movement, the energy consumed by the anchor node for collecting data, the energy used to travel to the next anchor node, and the energy supplied to anchor nodes. The mobile sink needs to use its energy by appropriately distributing it across these four items, and the amount can be determined by the total number of nodes, the number of anchor nodes, and the travel distance.

**Figure 4:**
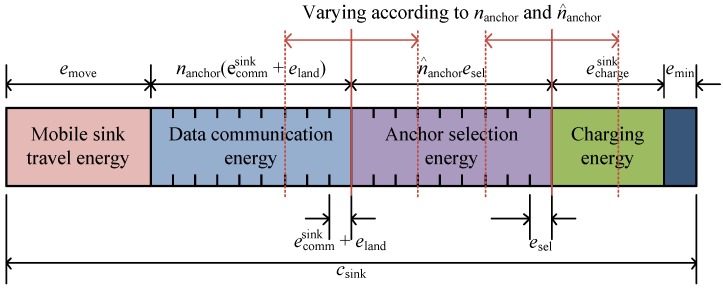
Energy model of the mobile sink.

The mobile sink visits the anchor nodes selected in the previous round, receives the data aggregated by each anchor node, and transfers energy to node. At this time, the drone lands at the anchor position because it must stop for a certain period of time to receive data. Therefore, for the drone to receive data from one anchor node, the energy for landing and takeoff, $e_{\mathrm{land}}$, and the average energy required for data communication, ${e_{}}_{\mathrm{comm}}^{\mathrm{sink}}$, is consumed. ${e_{}}_{\mathrm{comm}}^{\mathrm{sink}}$ can be changed depending on the communication range between the drone and the anchor nodes. It can be represented as follows:

(8) $${e_{}}_{\mathrm{comm}}^{\mathrm{sink}}=s_{\mathrm{control}}\beta {{r_{}}^{\mathrm{sink}}}^{\alpha }+{e_{}}_{\mathrm{Rx}}^{\mathrm{sink}},$$

where $s_{\mathrm{control}}$ is the length of the packets sent to require the aggregated data of anchor nodes, ${r_{}}^{\mathrm{sink}}$ is the transmission range of the drone, and ${e_{}}_{\mathrm{Rx}}^{\mathrm{sink}}$ is the energy consumed when receiving data. Therefore, when the number of anchor nodes is $n_{\mathrm{anchor}}$, the energy consumed by the drone for data communication with the anchor nodes becomes $n_{\mathrm{anchor}}\left(e_{\mathrm{land}}+{e_{}}_{\mathrm{comm}}^{\mathrm{sink}}\right)$. When the mobile sink traverses the network, it should select the nodes that will become anchor nodes in the next round and inform the next anchor nodes of this fact. When the energy consumed to inform the nodes of a new anchor node is defined as ${e_{}}_{\mathrm{sel}}$ and the number of anchor nodes in the next round is ${\hat{n_{}}}_{\mathrm{anchor}}$, the energy consumed in selecting new anchor nodes is ${\hat{n_{}}}_{\mathrm{anchor}}{e_{}}_{\mathrm{sel}}$. The detailed anchor selection process and how to calculate ${e_{}}_{\mathrm{sel}}$ are described in Section 3.3. The moving energy $e_{\mathrm{move}}$ has a constant value because the mobile sink moves along a fixed path determined by the network scale. The remaining energy in the mobile sink can then be used to charge the anchor nodes. Therefore, the entire energy capacity required for the mobile sink, $c_{\mathrm{sink}}$, can be expressed as follows:

(9) $$c_{\mathrm{sink}}=e_{\mathrm{move}}+n_{\mathrm{anchor}}\left(e_{\mathrm{land}}+{e_{}}_{\mathrm{comm}}^{\mathrm{sink}}\right)+{\hat{n_{}}}_{\mathrm{anchor}}{e_{}}_{\mathrm{sel}}+{e_{}}_{\mathrm{charge}}^{\mathrm{sink}}+{e_{}}_{\mathrm{sys}}^{\mathrm{sink}},$$

where ${e_{}}_{\mathrm{charge}}^{\mathrm{sink}}$ is the energy used to charge anchor nodes, and ${e_{}}_{\mathrm{sys}}^{\mathrm{sink}}$ is the energy consumed during standby and idle states in the drone. In Equation (9), because $e_{\mathrm{move}}$, $e_{\mathrm{land}}$, ${e_{}}_{\mathrm{comm}}^{\mathrm{sink}}$, and ${e_{}}_{\mathrm{sel}}$ are almost fixed values, the number of anchor nodes and the amount of charge energy they require represents a trade-off relationship. In the proposed scheme, Equation (9) is used to determine $n_{\mathrm{anchor}}$, ${\hat{n_{}}}_{\mathrm{anchor}}$, and ${e_{}}_{\mathrm{charge}}^{\mathrm{sink}}$, represented by $c_{\mathrm{sink}}$. Details on how to select anchor nodes are described in Section 3.4. Figure 4 shows the energy model of the mobile sink. In this figure, the data communication energy region and the anchor selection energy region can be changed by $n_{\mathrm{anchor}}$ and ${\hat{n_{}}}_{\mathrm{anchor}}$, and the size of the charging energy region is determined accordingly.

### 3.3. Mobile Sink Operations

**Figure 5:**
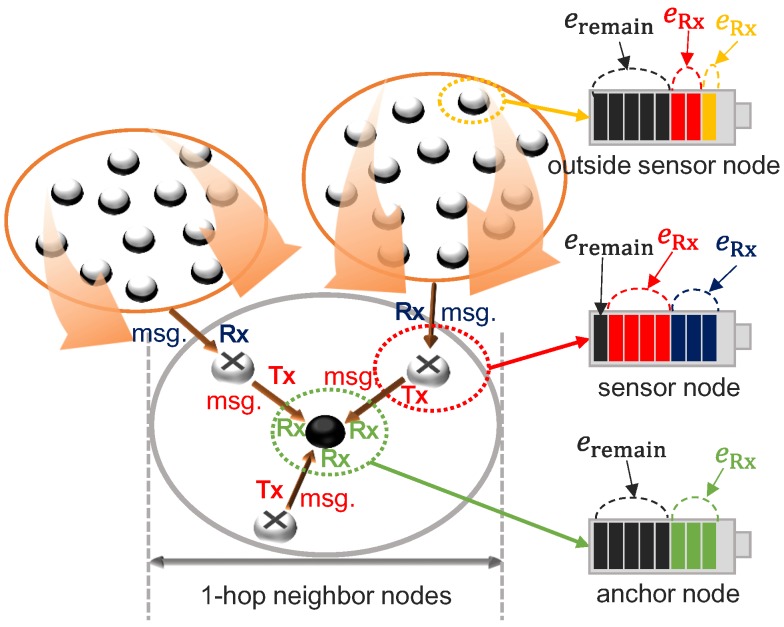
Energy states depending on node locations.

The mobile sink node performs mainly two tasks. The first task is to visit the existing anchor nodes to collect data. The sink node periodically traverses the path and collects the aggregated data for the last round at the anchor node. At this time, the sink node distributes its remaining energy, ${e_{}}_{\mathrm{charge}}$, to each anchor node, so that the anchor node does not fall into the outage state. The second task is to select new anchor nodes. The sink node moves to anchor candidate regions (the method to select anchor candidate regions will be described in Section 3.4 in detail) to select an anchor, and selects one of the sensor nodes in the region as an anchor node by broadcasting an *information request message* to the sensor nodes to request information of them. Anchor candidate nodes that received this message check whether they can survive until the next round when they become anchor nodes by considering Equations (5) and (6). Nodes that satisfy Equation (6) transmit their estimated remaining energy, ${\hat{e_{}}}_{\mathrm{remain}}^{\mathrm{anchor}}$, to the sink node. The sink node selects the node with the lowest ${\hat{e_{}}}_{\mathrm{remain}}^{\mathrm{anchor}}$ among the anchor candidate nodes as the anchor node. The reason is that although the anchor node consumes the most energy while collecting the data and sending it to the sink, if the energy state of the anchor node satisfies Equation (6), the needed energy is supplied through the drone to prevent it from blackout. In addition, because the neighboring nodes of the anchor node consume a large amount of energy, it is necessary to have more energy than other nodes to prevent blackout and thus reduce data loss, as shown in Figure 5.

**Figure 6:**
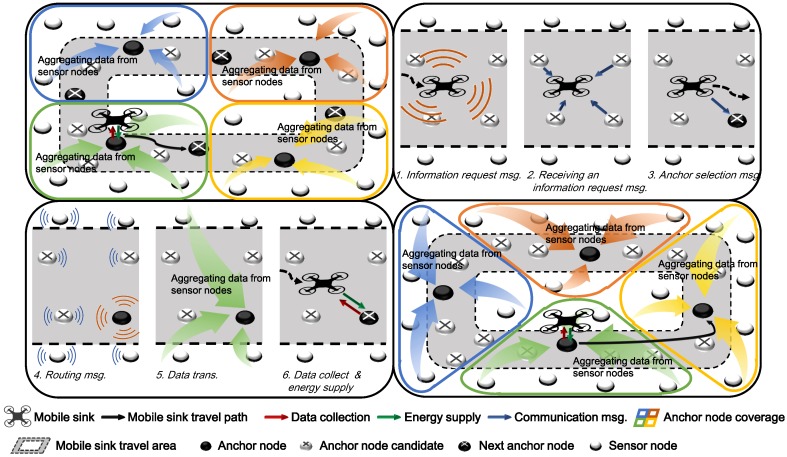
Anchor node selection process.

Therefore, the sink node selects an anchor node that satisfies Equation (6) and has the lowest energy among the anchor candidates, and it sends an *anchor notification message* to this node to appoint it as the anchor in the next round. Figure 6 shows the overall process of selecting an anchor node. While the drone performs this process after arriving at an anchor candidate region, the drone broadcasts *information request message* and receives responses to it. After selecting an anchor, it broadcasts *anchor notification message* to inform which node is the anchor. The energy consumed in this process, ${e_{}}_{\mathrm{sel}}$ can be expressed as follows:

(10) $${e_{}}_{\mathrm{sel}}={e_{}}_{\mathrm{sel}}^{\mathrm{req}}+{e_{}}_{\mathrm{sel}}^{\mathrm{info}}+{e_{}}_{\mathrm{sel}}^{\mathrm{noti}},$$

where ${e_{}}_{\mathrm{sel}}^{\mathrm{req}}$ is the energy consumed to broadcast *information request message*, ${e_{}}_{\mathrm{sel}}^{\mathrm{info}}$ is the energy consumed in receiving a response to the *information request message*, and ${e_{}}_{\mathrm{sel}}^{\mathrm{noti}}$ is the energy consumed broadcast the *anchor notification message* to inform which node is the anchor.

After the drones have collected data and have finished traversal, the drone must be recharged for the next round. However, the charging time of the drone can take several hours (for a 3950 mhA battery, more than 1.5 h [46]). As a result, if the duration of each round is short, the drone may not be sufficiently charged. Methods to solve this problem are to (i) configure the duration of a round longer than the charging time, (ii) use several drones alternately, and (iii) use several replaceable batteries.

### 3.4. Anchor Node Selection and Data Collection

The number of hops of a sensor node can be changed according to the location of its anchor node, which is the destination for its data. Therefore, it is important to select the number and location of anchor nodes efficiently to reduce the energy consumption of the sensor nodes. To minimize the number of hops of a sensor node, the number of anchor nodes during a round should be maximized and arranged evenly. Because the anchor nodes are selected along a predetermined path, if the number of anchors is fixed, selecting a certain number of anchors at a fixed distance interval on the path will minimize the data route length(the number of hops) of the sensor nodes. Therefore, in this scheme, the sink traverses the specified path, and arranges the anchor nodes uniformly at regular intervals starting from a random location.

It is necessary to determine the number of anchor nodes capable of collecting the data of all sensor nodes and of transferring the data to the sink using the energy provided by the sink. The anchor nodes transmit data only when the sink visits but consume significant energy at that moment. Therefore, to prevent and anchor node from blackout during data transmission, when a sink arrives at an anchor node, the sink should transmit sufficient energy to the anchor node so that the anchor node successfully transmits all data. In this case, the sensor node might not have enough energy to transmit all data, but it can transmit data with energy transferred from the mobile sink. The sensor node waits without transmitting data until it receives enough energy to transmit data. Assuming that the amount of data that the sensor node transmits is *s* during one round, which can be varied depending on the applications, and the sensor and anchor nodes are evenly distributed, then $\frac{n_{}}{n_{\mathrm{anchor}}}s$ data should be transmitted. The average amount of energy required to transmit is determined from Equation (3) as follows:

(11) $$\frac{n_{}}{n_{\mathrm{anchor}}}s\beta {r_{}}^{\alpha }.$$

The sink node should transmit at least this amount of energy to the anchor node. Therefore, the minimum amount of energy that needs to be transmitted to all anchors in one round, ${e_{}}_{\mathrm{charge}}^{\min}$, can be calculated as follows:

(12) $${e_{}}_{\mathrm{charge}}^{\min}=n_{\mathrm{anchor}}\frac{n_{}}{n_{\mathrm{anchor}}}\frac{s\beta {r_{}}^{\alpha }}{\eta }=\frac{n_{}s\beta {r_{}}^{\alpha }}{\eta },$$

where η is the energy transfer efficiency. In the proposed scheme, because the size of the network does not change and the sensor nodes periodically collect the same amount of data, the number of anchor nodes does not change drastically. Therefore, with $n_{\mathrm{anchor}}$ essentially equal to ${\hat{n_{}}}_{\mathrm{anchor}}$, and the minimum value of ${e_{}}_{\mathrm{charge}}$ is ${e_{}}_{\mathrm{charge}}^{\min}$, Equation (9) can be rewritten as follows:

(13) $$c_{\mathrm{sink}}\geq e_{\mathrm{move}}+n_{\mathrm{anchor}}\left(e_{\mathrm{land}}+{e_{}}_{\mathrm{comm}}^{\mathrm{sink}}+{e_{}}_{\mathrm{sel}}\right)+{e_{}}_{\mathrm{charge}}^{\min}+{e_{}}_{\mathrm{sys}}^{\mathrm{sink}}.$$

If this expression is solved in terms of $n_{\mathrm{anchor}}$, it can be expressed as follows:

(14) $$n_{\mathrm{anchor}}\leq \frac{c_{\mathrm{sink}}-e_{\mathrm{move}}-{e_{}}_{\mathrm{charge}}^{\min}-{e_{}}_{\mathrm{sys}}^{\mathrm{sink}}}{e_{\mathrm{land}}+{e_{}}_{\mathrm{comm}}^{\mathrm{sink}}+{e_{}}_{\mathrm{sel}}}.$$

Because we must select as many anchor nodes as possible to mitigate energy imbalances and hotspots, the number of anchor nodes, $n_{\mathrm{anchor}}$, is determined as follows:

(15) $$n_{\mathrm{anchor}}=\left\lfloor \frac{c_{\mathrm{sink}}-e_{\mathrm{move}}-{e_{}}_{\mathrm{charge}}^{\min}-{e_{}}_{\mathrm{sys}}^{\mathrm{sink}}}{e_{\mathrm{land}}+{e_{}}_{\mathrm{comm}}^{\mathrm{sink}}+{e_{}}_{\mathrm{sel}}}\right\rfloor .$$

In addition, based on the $n_{\mathrm{anchor}}$ derived here, ${e_{}}_{\mathrm{charge}}$ is recalculated by substituting $n_{\mathrm{anchor}}$ and ${\hat{n_{}}}_{\mathrm{anchor}}$ into Equation (9), through which the amount of energy that is to be distributed to each anchor node, $e_{\mathrm{charge}}/n_{\mathrm{anchor}}$, can be calculated.

When the sink node travels through a predetermined path and visits the anchor nodes to collect the aggregated data, it supplies the energy, $e_{\mathrm{charge}}/n_{\mathrm{anchor}}$, to each anchor node to prevent power failure. If more energy cannot be transmitted because it exceeds the battery capacity of the node, the remaining energy can be distributed to the following anchor nodes.

### 3.5. Routing and Data Collection

After an anchor node is selected, it broadcasts a *routing message* including its ID as the anchor node ID along the maximum number of hops to inform other sensor nodes that it is their anchor node, ordering the nodes to determine a routing path. A sensor node that receives this message accepts the node that has the anchor ID as its anchor node, records the anchor ID, the hop count to the anchor node, and its own node ID, and rebroadcasts this message. During this process, the maximum number of hops in this message is limited to prevent it from being transmitted to too many nodes, so the maximum hop number is decremented by 1 after each hop, and the message is not retransmitted once this count reaches 0. If a sensor node has already determined its anchor node but receives another *routing message*, then the number of hops to the previously recorded anchor is compared with the number of hops to the new anchor, and the anchor requiring fewer hops is reconfigured as its anchor.

**Figure 7:**
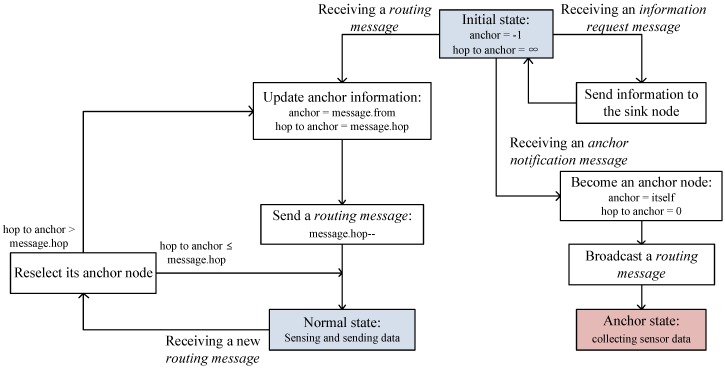
Flowchart of anchor selection.

All sensor nodes transmit their data to the selected anchor and the transmission path becomes the reverse path of the *routing message*. However, if new anchor nodes are selected during data transfer, data can be transferred to the previous anchors. In this case, the previous anchors store it and then, send it to the new anchors with their own sensing data when they receive the *routing messages* from the new anchors. Figure 7 depicts the anchor selection and routing process flowchart.

We have described how to determine the number of anchor nodes and how to manage and select the following anchor nodes when the mobile sink traverses a path to collect data. Applying this scheme to an energy-harvesting WSN can prevent node blackouts and reduce hotspot problems. Additionally, when data is collected from an anchor node, transferring available energy from the sink to the anchor nodes, which consume significant energy during data transmission, can enable successful continuity of data transmissions without power failures; thus, the proposed method can increase the data collection rate.

## 4. Performance Evaluation

### 4.1. Simulation Environment

We have compared the performance of our scheme with others: (i) anchors with fixed positions with WPT (*fixed*); (ii) anchors with random positions with WPT (*random*); (iii) randomly positioned anchors with no WPT (*random (no charging)*). All schemes except for the proposed scheme select anchor nodes according to a fixed number of anchor nodes. The *fixed* and *random* schemes fully charge the batteries of the anchor nodes when the drone arrives. The characteristics of the comparison schemes are as follows:

- *Fixed*: In this scheme, the constant number of anchors are selected on the predefined locations. The drone collects data at a predefined location each time. The drone distributes $\frac{{e_{}}_{\mathrm{charge}}}{n_{\mathrm{anchor}}}$ energy to each anchor by dividing ${e_{}}_{\mathrm{charge}}$ equally by the number of anchors. At this time, if the transferred energy exceeds the amount that the node can store, the energy is no longer transferred, and the extra energy is distributed to the non-visiting anchors.
- *Random*: In this scheme, the drone selects the constant number of anchors every round. The position of the anchors changes randomly within the travel path of the drone every round to alleviate the energy imbalance problem. Like the *fixed* scheme, the drone distributes energy by dividing ${e_{}}_{\mathrm{charge}}$ equally by the number of anchors, and the extra energy is distributed to the non-visiting anchors.
- *Random (no charging)*: This scheme is identical to the *Random* scheme except that the drone does not transfer energy. We chose this scheme for comparison with the scheme that does not use WPT.

We measured the change in the amount of data collected at the mobile sink, the amount of data sensed at the sensor nodes, and the number of blackout nodes based on the number of sensor nodes and anchor nodes and the density change. Each test set was run 30 times over 60 days to obtain the average values. Table 1 shows the important parameters used in the simulation.

### 4.2. Simulation Results

#### 4.2.1. Performance Evaluation over Time

**Figure 8:**
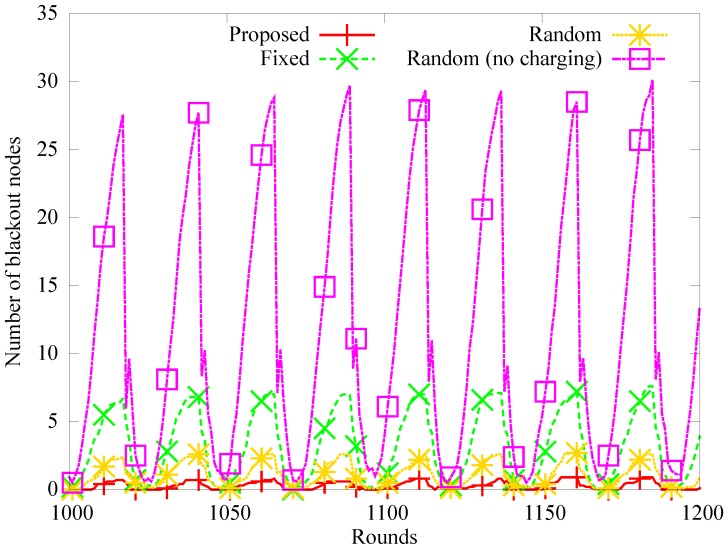
Change in the number of blackout nodes.

**Figure 9:**
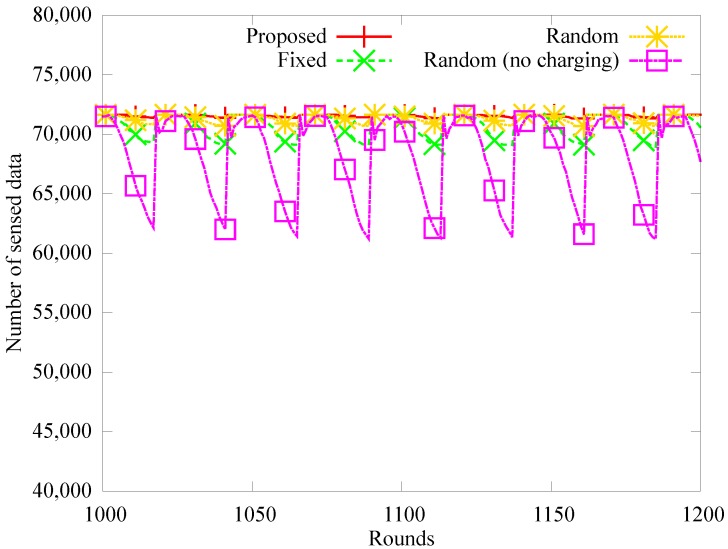
Comparison of amount of sensed data.

**Figure 10:**
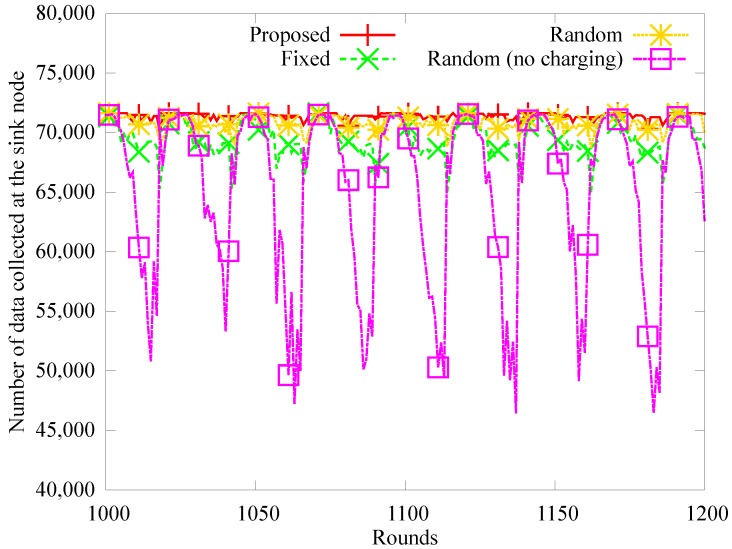
Comparison of amount of gathered data.

Figure 8, Figure 9 and Figure 10 show the number of blackout nodes, the quantity of data sensed, and the quantity of collected data for approximately eight days (from 1000 to 1200 rounds) with node density of 0.04. Because the sensor nodes use solar energy, the number of blackout nodes is low during the daytime, but the energy is not recharged at night, so the number of blackout nodes increases in Figure 8. Specifically, the *random (no charging)* scheme that does not use WPT, experiences a higher number of blackouts than the other schemes during nighttime. In the *fixed* method, the position of the anchor nodes does not change even though they can be charged with WPT, therefore, blackouts occur more frequently owing to the hotspot problem. In the proposed and *random* scheme, the number of anchor nodes and the amount of charging energy are appropriately adjusted. However, fewer blackouts occur in the proposed scheme than in the *random* scheme. The amount of sensed data decreases as the number of blackout nodes increases, as shown in Figure 9. On the other hand, in Figure 10, the amount of data collected by the mobile sink node is much lower than the amount of sensed data in the random scheme, because as shown in Figure 9, many relay nodes around the anchor nodes experience blackouts owing to the hotspot problem.

#### 4.2.2. Performance According to the Number of Nodes

**Figure 11:**
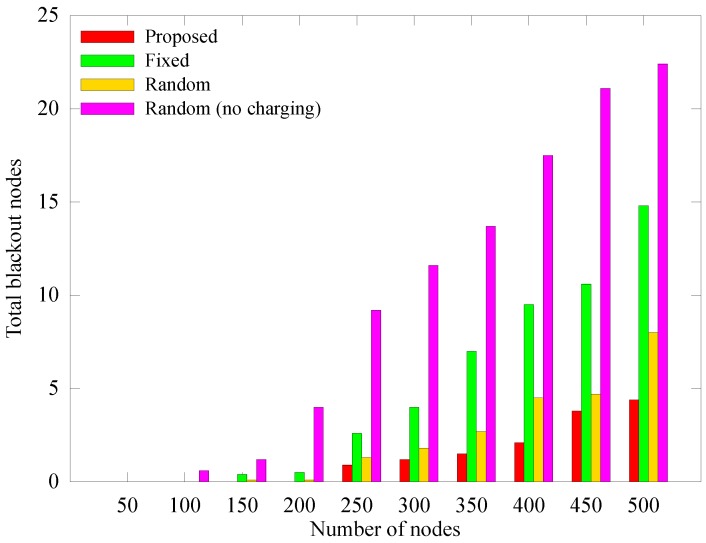
Comparison of in the number of blackout nodes according to the number of nodes.

**Figure 12:**
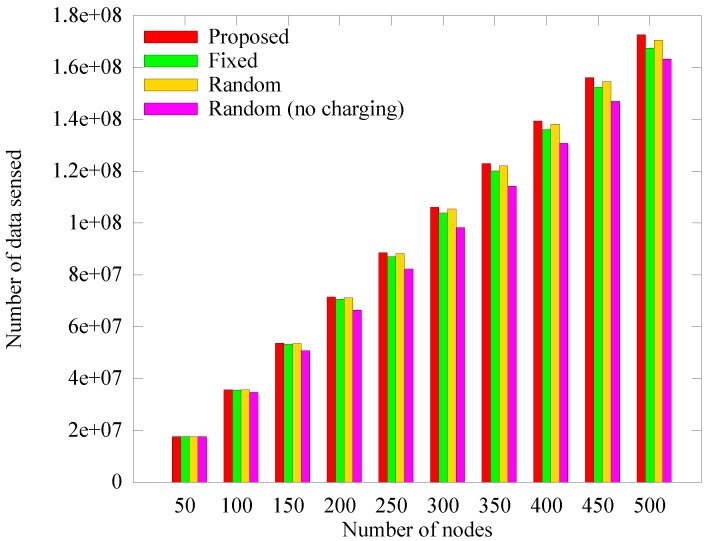
Comparison of in the quantity of data sensed according to the number of nodes.

**Figure 13:**
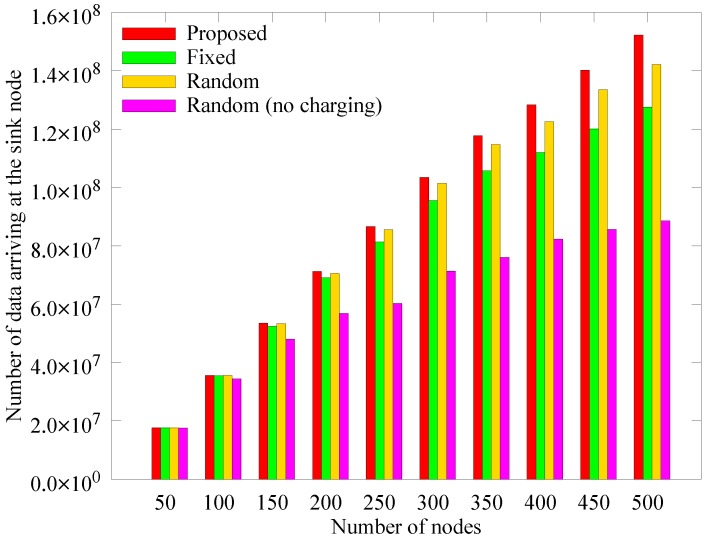
Comparison of the quantity of data collected at the mobile sink node according to the number of nodes.

Figure 11, Figure 12 and Figure 13 respectively depict a comparison of the number of blacked out nodes, data collected by the sink, and sensed data according to the number of nodes, which is varied from 50 to 500 in 50 unit intervals with node density of 0.04. These results are measured at 1000 rounds. In Figure 11, as the number of nodes increases, an anchor node must aggregate more data, which leads the hotspot problem. As a result, the number of blackout nodes increases. In the *random (no charging)* scheme which does not use WPT, the number of blackout nodes increases drastically because the anchor nodes cannot handle all the transmitted data. In the *fixed* scheme, even though the energy required to select the anchors is saved because the anchor nodes are fixed, the blackout of anchor nodes rapidly increases owing to the hotspot problem. In contrast, the proposed scheme is less affected by the number of nodes. The reason is that the proposed scheme appropriately selects the numbers and locations of anchors and determines the proper amount of energy to deliver. As discussed in Section 4.2.1, Figure 12, illustrates the increase in the amount of sensed data as the number of nodes increases, and Figure 13 shows that the amount of data collected does not increase significantly despite the increases in blackouts. However, the proposed scheme can adaptively collect larger amounts of data, regardless of the number of nodes.

#### 4.2.3. Performance According to the Node Density

**Figure 14:**
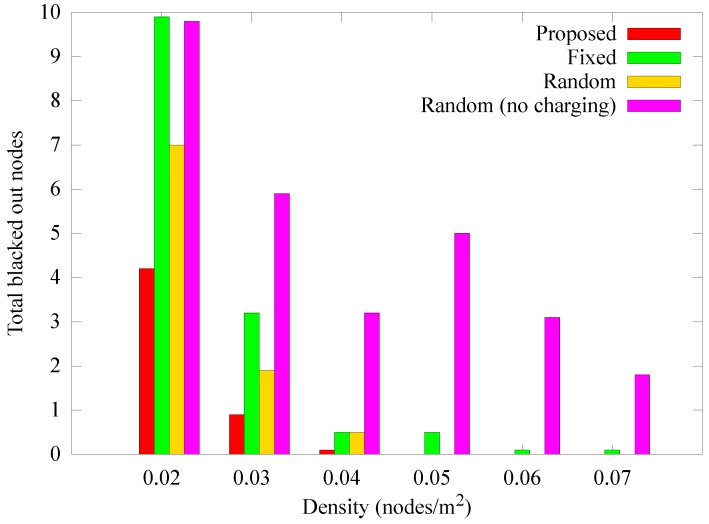
Comparison of the number of blackout nodes according to the density.

**Figure 15:**
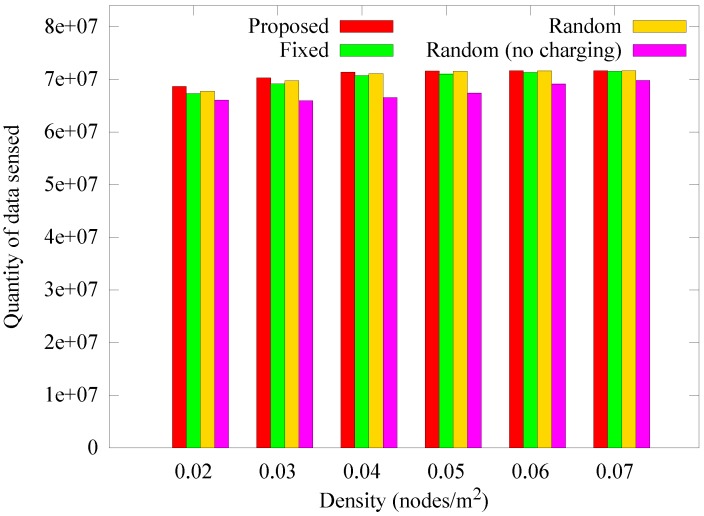
Comparison of the amount of sensed data according to the density.

**Figure 16:**
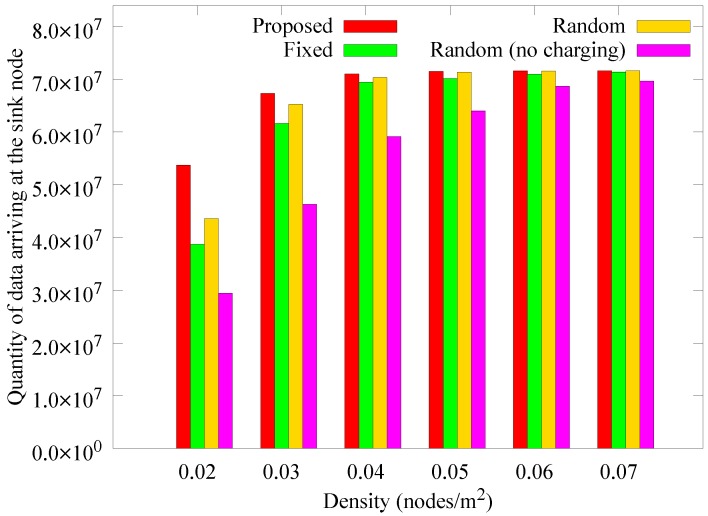
Comparison of the quantity of data collected at the mobile sink node according to the density.

Figure 14 presents the change in the number of blackout nodes according to the node density at 1000 rounds. When the density is low, relay nodes consume more energy because the hop count between the sensor nodes and the anchor node increases. As a result, blackouts occur more frequently as density is decreased. If the density is increased, more nodes can deliver data to the anchor nodes in one hop. In addition, because the anchor nodes transmit data only when the mobile sink arrives, the number of blackouts decreases because the hotspot problem is reduced. However, in the *random (no charging)* scheme, the anchor nodes cannot obtain energy from a mobile sink, resulting in higher energy consumption, thereby increasing the number of blackouts. Figure 15 and Figure 16 show how the quantity of data sensed and the quantity of data received by the sink node change according to density. As in the previous case, when the density is low, the amount of data collected by the sink is less than the data sensed owing to the influence of blackouts. Nevertheless, the proposed scheme experiences less data reduction for low node densities than the other schemes, and is less affected by density.

#### 4.2.4. Performance According to the Number of Anchor Nodes

**Figure 17:**
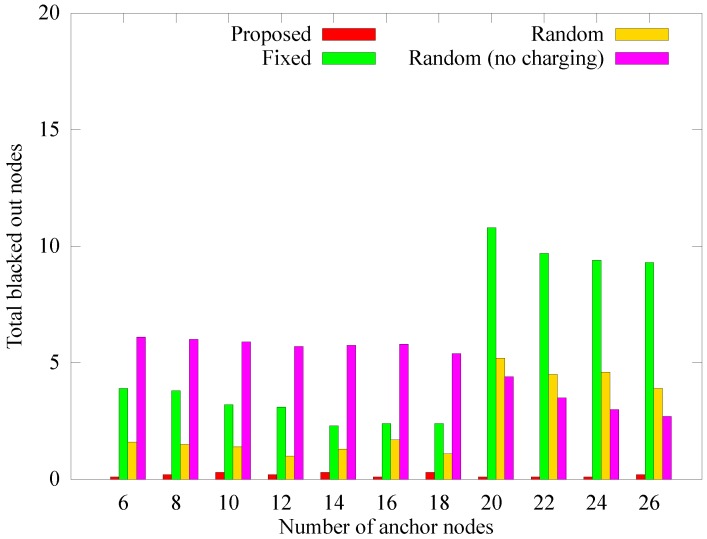
Comparison of the number of blackout nodes according to the number of anchor nodes.

**Figure 18:**
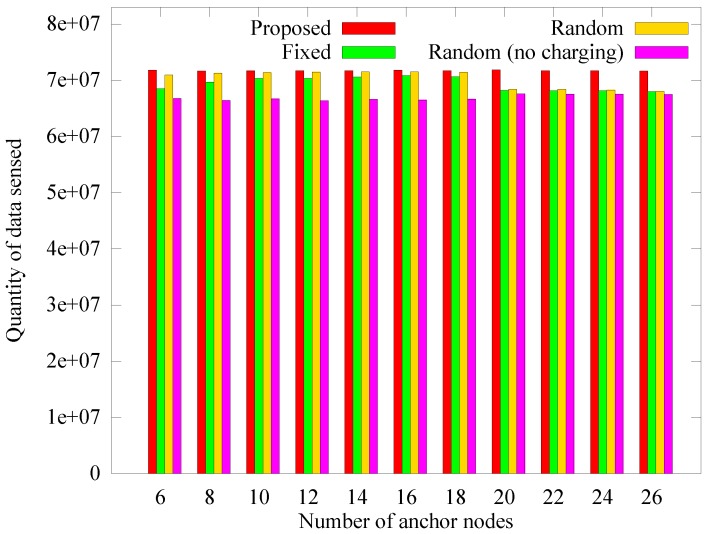
Comparison of the quantity of data sensed according to the number of anchor nodes.

**Figure 19:**
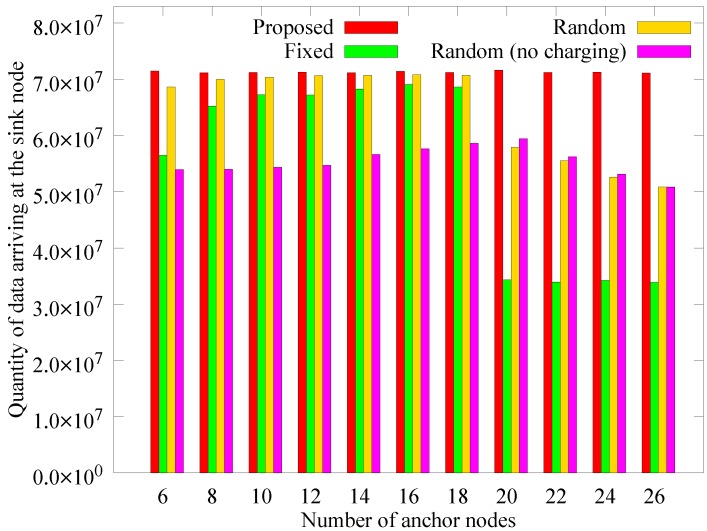
Comparison of the quantity of data collected at the mobile sink node according to the number of anchor nodes.

The number of anchor nodes is fixed in other schemes, while the proposed method adaptively determines the number of anchor nodes. We measured the performance of the different schemes according to the change in the number of anchors to verify whether the proposed scheme selects the appropriate number of anchor nodes. In the proposed scheme, the number of anchor nodes calculated dynamically is 18 on average, and the numbers of anchor nodes in Figure 17, Figure 18 and Figure 19 apply only to the other schemes. Figure 17 shows the change in the number of blackout nodes as the number of anchor nodes increases. As the number of anchor nodes increases, the number of blackout nodes decreases. This is because the hotspot problem is mitigated by diversifying the anchors for each node. However, if the number of anchor nodes becomes greater than 18, the blackout nodes of the fixed and random scheme increase sharply (the proposed scheme chooses 18 dynamically). This phenomenon is caused by a lack of energy available for transfer to the anchor nodes due to the energy losses incurred by frequent landings and takeoffs. As the landing energy increases, the rear anchor nodes blackout because the remaining energy in the sink has been transferred to only some nodes earlier or no one can receive the energy at all, leading to a similar performance as for the *random (no charging)* scheme.

However, as the number of anchors increases from 20 to 26, the number of blacked out nodes decreases. This phenomenon is because the transmission hop count of sensor nodes is reduced due to the distributed anchors. Figure 18 and Figure 19 respectively present the change in the quantity of sensed data and the quantity of data received at the sink as the number of anchor nodes changes. Similar to when the number of blackout nodes increases, when the number of anchor nodes increases, the quantity of collected data gradually increases, and when the number of anchors exceeds a limit, the quantity of collected data decreases rapidly. This is because, similar to the comparison of the number of blackout nodes, the mobile sink has no available energy left to charge the rear anchor nodes. The proposed scheme determines the number of anchor nodes according to the network environment. In the *random* scheme, when the number of anchor nodes reaches 18, the number of blacked out nodes reaches its minimum. This result supports our argument that the proposed scheme dynamically determines the proper number of anchor nodes.

We have verified the performance of the proposed scheme through the various simulations and comparisons. As a result, the proposed scheme exhibits better performance than other methods by uniformly collecting sensory data despite changes in the numbers of sensor and anchor nodes or in proper the node density. This is because it determines the appropriate number of anchors and the proper amount of energy to be transferred to the anchor nodes according to the size of the network, the number of nodes, the amount of data sensed, and the amount of energy to be consumed.

## 5. Conclusions

We proposed a scheme to increase network connectivity and the quantity of data collected in energy-harvesting WSNs by using a mobile sink that has WPT capabilities. In the proposed scheme, anchor nodes were selected by considering the energy capacity of the mobile sink, the size of the network, the amount of data to be collected, and the energy state of the nodes, where a certain quantity of anchor nodes aggregate data from other sensor nodes. While the mobile sink node visits anchor nodes to collect the accumulated data, it transfers energy directly to each anchor node simultaneously. This scheme mitigates the hotspot problem by dynamically selecting the maximum possible number of anchor nodes, through which the number of hops and the relay energy consumed by the nodes are reduced. Additionally, anchor nodes, which consume significant energy when transmitting, receive energy directly from the mobile sink, thus preventing blackouts during transmission and improving the network’s sustainability. Consequently, this technique reduces data losses compared to other schemes, leading to an increased amount of collected data. In the future, we plan to study a scheme that further increases the amount of data collected by controlling the amount of sensing data produced by the energy-rich outer nodes.

## Figures and Tables

**Table 1:** Simulation parameters.

Parameter	Value
Number of nodes	200
Node density	0.04
Simulation Time	60 days
Deploy	Random
Routing	MDT
Radio Range	10 m
Battery capacity	110 mAh
Round	1 h
Mobile sink battery capacity	4480 mAh
Max. mobile sink moving distance	2000 m
WPT efficiency	50%
Number of anchor nodes	18
